# Dynamic contact angle of water-based titanium oxide nanofluid

**DOI:** 10.1186/1556-276X-8-282

**Published:** 2013-06-11

**Authors:** Milad Radiom, Chun Yang, Weng Kong Chan

**Affiliations:** 1 Department of Chemical Engineering, Virginia Tech, Blacksburg, VA 24060, USA; 2 School of Mechanical and Aerospace Engineering, Nanyang Technological University, 50 Nanyang Avenue, Singapore 639798, Singapore

**Keywords:** Dynamic contact angle, Hydrodynamic theory, Molecular kinetic theory, Nanofluids, Nanoparticles, Non-Newtonian fluid, 68.08.Bc

## Abstract

This paper presents an investigation into spreading dynamics and dynamic contact angle of TiO_2_-deionized water nanofluids. Two mechanisms of energy dissipation, (1) contact line friction and (2) wedge film viscosity, govern the dynamics of contact line motion. The primary stage of spreading has the contact line friction as the dominant dissipative mechanism. At the secondary stage of spreading, the wedge film viscosity is the dominant dissipative mechanism. A theoretical model based on combination of molecular kinetic theory and hydrodynamic theory which incorporates non-Newtonian viscosity of solutions is used. The model agreement with experimental data is reasonable. Complex interparticle interactions, local pinning of the contact line, and variations in solid–liquid interfacial tension are attributed to errors.

## Background

Industrial operations such as spin coating, painting, and lubrication are based on spreading of fluids over solid surfaces. The fluid may be simple [1-3] or particulate such as paint, ink, or dye [4]. For many years, capillary flow of simple fluids has received considerable attention, and physics of capillary action is known for a long time [5-9]. In addition, capillary flow of micellar surfactant solutions which contain monodisperse and naturally stabilized nanoparticles has been studied [10-14]. However, the same study on liquids laden with metallic and oxide nanoparticles such as silver, copper, zinc oxide, and titanium oxide is scarce. These fluid suspensions are termed as nanofluids after the seminal work by Choi and Eastman [15]. The application of nanofluids is coined with enhanced heat transfer performance compared with their base fluids. They are proposed for applications in cooling of electronic devices, ventilation and air conditioning, and biomedical applications [14,16-24].

It is known that out-of-equilibrium interfacial energy (*σ*(cos *θ*^0^ − cos *θ*)) provides free energy of capillary flow where *σ* is the liquid-air surface tension and *θ*^0^ and *θ* are the equilibrium and dynamic contact angles, respectively. During capillary flow, the free energy is dissipated by two mechanisms [5]: (1) contact line friction (*T* ∑ _*l*_) which occurs in proximity of three-phase contact line (solid–liquid–air). The friction at the three-phase contact line is due to intermolecular interactions between solid molecules and liquid molecules. (2) Wedge film viscosity (*TΣ*_*W*_) which occurs in the wedge film region behind the three-phase contact line. Lubricating and rolling flow patterns in the wedge film region result in the dissipation of the free energy. For each mechanism of energy dissipation, a theory is developed: (1) molecular kinetic theory (MKT) [25,26] models the contact line friction, and (2) hydrodynamic theory (HDT) [27,28] models the wedge film viscosity. For partial wetting systems (*θ*^0^ > 10*°*), it is assumed that both dissipative mechanisms coexist and models that combine MKT and HDT are developed by Petrov [29] and De Ruijter [30]. In Petrov's model, it is assumed that the equilibrium contact angle *θ*^0^ is not constant and its change is described by MKT. In De Ruijter's model, it is assumed that *θ*^0^ is constant and the dissipation functions are added to form the total dissipation function, *TΣ*_tot_ = *T* ∑ _*l*_ + *TΣ*_*W*_. These models are developed for Newtonian fluids and show generally good agreement with experimental data [31].

This paper presents an investigation into spreading dynamics and dynamic contact angle of TiO_2_-deionized (DI) water nanofluids. Metal oxide TiO_2_ nanoparticle was chosen for its ease of access and popularity in enhanced heat removal applications. Various nanoparticle volume concentrations ranging from 0.05% to 2% were used. The denser solutions exhibit non-Newtonian viscosity at shear rate ranges that are common to capillary flow. To model experimental data a theoretical model based on combination of MKT and HDT similar to De Ruijter's model is used. The non-Newtonian viscosity of the solutions is incorporated in the model.

## Methods

### Preparation of nanofluids

**Figure 1:**
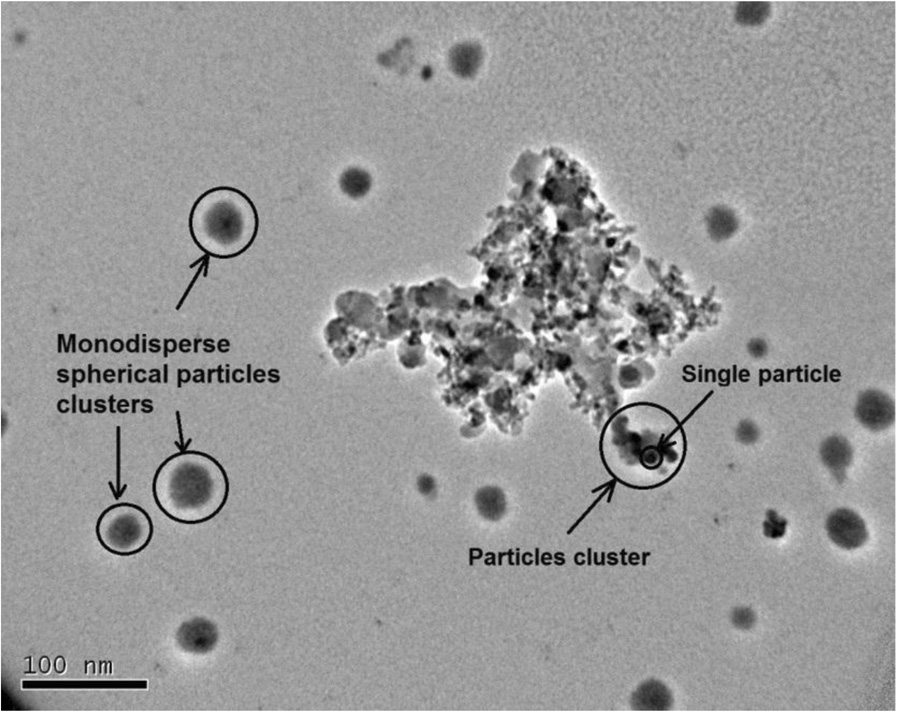
**TEM nanographs of 15 nm TiO**_**2**_**nanoparticles.**

The solutions were prepared by dispersing 15 nm TiO_2_ nanoparticles (anatase, 99%, Nanostructured and Amorphous Materials Inc., Houston, TX, USA) in DI water. Oleic acid is reported to stabilize TiO_2_ nanoparticles in DI water [20] and was added to the mixture at 0.01vol.% concentration. The solution was stirred for 8 h followed by 100 min sonication (Sonicator 3000, 20 kHz and 80 kW, MISONIX, Farmingdale, NY, USA). Temperature of the solution was maintained at 25°C during the sonication process. Clustering and morphology of nanoparticles are important factors in nanofluid spreading capability. We used transmission electron microscopy (TEM) nanographs of TiO_2_ nanoparticles to examine these factors. An aliquot of dilute solution was dropped and dried on a carbon-coated copper grid. TEM images were then taken immediately. Figure 1 shows that the solution contains irregular particle clusters in addition to monodispersed particles. The sizes of the single particles were found to be close to 15 nm as specified by the supplier. The morphology of the monodispersed particles is spherical. Sonication of the nanofluid solution and addition of surfactant molecules is critical to break down the particle agglomerations and stabilize particle dispersion. The effective nanoparticle size was 260 nm measured with a particle size analyzer (Brookhaven Instruments Corporation, Holtsville, NY, USA). Adsorption of oleic acid surfactant molecules to the surface of TiO_2_ particles and dissociation of proton from carboxylic acid head groups result in net negative charges on the surface of particles and thus formation of electric double layer around them. Thick electric double layers cause the deviation of particle-particle interactions from hard-sphere interactions. The (Debye) length in nanometer of an electric double layer of 1:1 electrolyte in water at 25°C can be approximated by $0.3/\sqrt{M}$ (where *M* is the molar concentration). For 0.01 vol.% concentration of oleic acid in water (which is 3.15 × 10^-4^ molar), the Debye length is estimated to be about 16.9 nm. Such a small increase in the effective diameter of particles allows for an assumption of hard-sphere interactions between particles in the solution which is an important assumption in using Krieger's formula [32]. All other experimental measurements were carried out at 25°C.

### Measurement of viscosity

Viscosity of the solutions was measured using a controllable low shear rate concentric cylinders rheometer (Contraves, Low Shear 40, Zurich, Switzerland). The viscosity was measured at shear rates ranging from 0 to 50 s^−1^. This range corresponds to the shear rates that are common to capillary flow.

### Measurement of surface tension

Surface tension of the solutions was measured by pendant droplet method using FTA200 system (First Ten Angstroms, Inc., Portsmouth, VA, USA). To form the pendant droplets, the solutions were pumped out of a syringe system at a very low rate, namely 1 μl/s, to minimize inertia effects. To minimize errors due to evaporation, surface tension was measured right after the pendant droplet reached its maximum volume, namely 10 μl for the dense solutions.

### Measurement of dynamic contact angle

**Figure 2:**
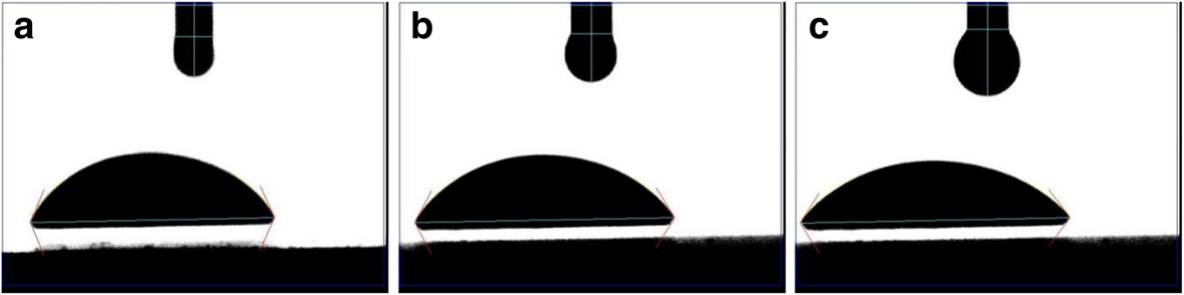
Consecutive photographs of spreading droplet detached from syringe needle tip.

Dynamic contact angle of the solutions was measured using the FTA200 system. A droplet of solution was generated at a very low rate (1 μl/s) and detached from the syringe needle tip as soon as it touched the borosilicate glass slide. A video was captured while the droplet was spreading over the glass slide from initial contact to equilibrium position (see Figure 2: the time frame elapses between (a) to (b) and (b) to (c) are 5 and 100 s, respectively). The consecutive photographs were used to measure the contact angles. The spatial resolution was estimated to be about 50 μm on the basis of the focused area and camera pixel size. The standard deviation for contact angle measurements was less than 1°. The temporal resolution was estimated based on the frame speed of the CCD camera as 30 fps. For each concentration, three experiments were performed and average was taken.

### Theory

#### Empirical analysis of viscosity

**Figure 3:**
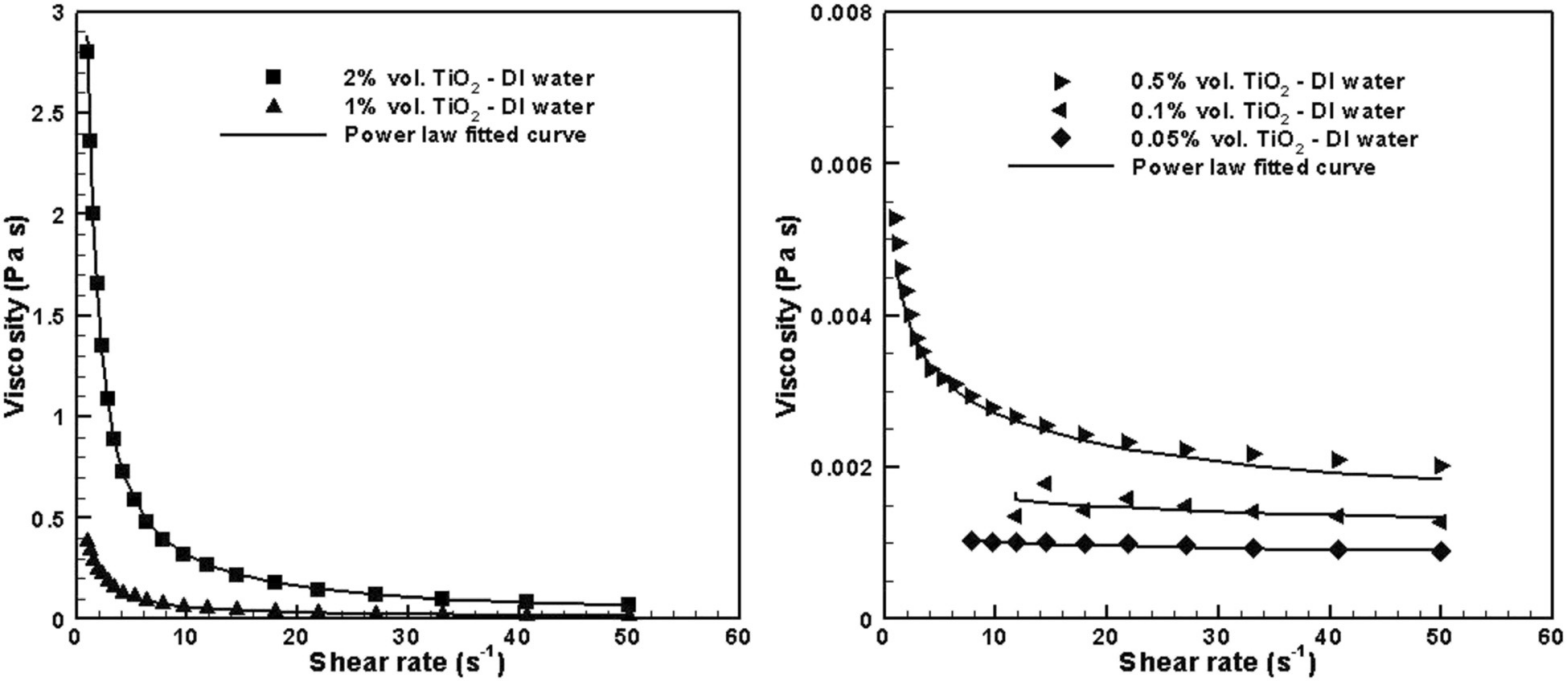
**Viscosity of TiO**_**2**_**-DI water solutions.**

From Figure 3, it is obvious that 0.5%, 1%, and 2% solutions exhibit shear thinning viscosity at shear rates below 20 s^−1^. At higher shear rates, Newtonian behavior was observed for all solutions. For dilute solutions, 0.1 vol.% and 0.05 vol.%, a weak shear thinning behavior was also observed at very low shear rates [19].

A power-law equation is used to model the shear rate and nanoparticle concentration dependent viscosity:

(1) $$\frac{\eta_{n}}{\eta_{b}}=F\left(\phi \right)K{\dot{\gamma }}^{n-1}$$

where *η*_*b*_ is the viscosity of DI water equal to 0.927 mPa s, *F*(*ϕ*) is a function of nanoparticle volume concentration (*ϕ*), $K{\dot{\gamma }}^{n-1}$ is an indicator of shear thinning viscosity with *K* as the proportionality factor, and *n* as the power-law index. *F*(*ϕ*) is calculated using Krieger's formula [32]:

(2) $$F\left(\phi \right)={\left(1-\frac{\phi }{\phi_{\max}}\right)}^{-2.5\phi_{\max}}$$

where *ϕ*_max_ is the fluidity limit that is empirically equal to 0.68 for hard spherical particles. In Equation 1, *n* and *K* are empirical constants which are obtained by fitting this equation to the experimental data shown in Figure 3. Table 1 shows the values of *K* and *n* for various nanoparticle volume concentrations. It is obvious that higher nanoparticle concentration results in a larger non-Newtonian behavior. Figure 3 also shows that the power-law Equation 1 is in good agreement with the experimental data.

**Table 1:** **Power-law viscosity, surface tension, and equilibrium contact angle of TiO**_**2**_**-DI water solutions**

**TiO**_**2**_**volume concentration (***ϕ***)**	**Power-law index (*n*)**	**Proportionality factor (*K*)**	**Surface tension (*σ*[*N*/*m*])**	**Equilibrium contact angle (*θ***^0^**)**
2%	0.04	2,932	0.0543	51.7
1%	0.18	432	0.0606	47.5
0.5%	0.76	5	0.0612	46.7
0.1%	0.89	2	0.0623	45.7
0.05%	0.92	1	0.0632	44.5

#### Molecular kinetic theory

**Figure 4:**
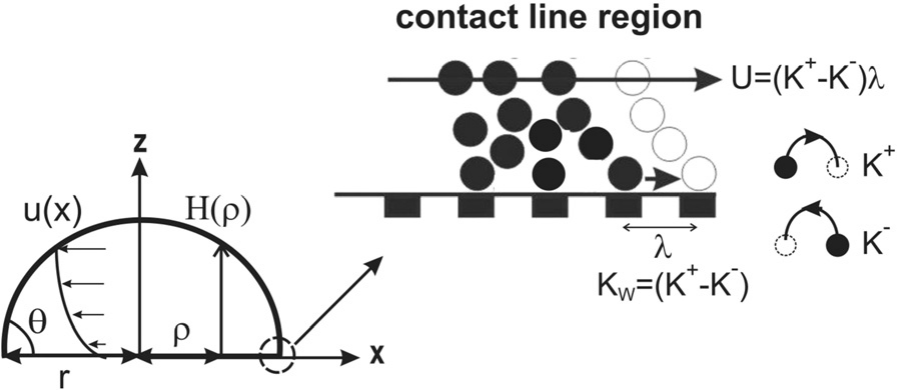
Schematic of a spreading droplet.

Schematic of a spreading droplet of radius *r* and contact angle *θ* that is inspired by De Gennes [5] and Blake [26] is depicted in Figure 4. Based on MKT [26], the rate of displacement of the three-phase contact line over adsorption sites on solid surface, *U*, is equal to the net frequency of molecular movements, *K*_*W*_ (*K*_*W*_ = *K*^+^ − *K*^−^, where *K*^+^ is the frequency of forward motion and *K*^−^ is the frequency of backward motion), multiplied by average distance between the adsorption sites, *λ*:

(3) $$U=\lambda K_{W}$$

The equilibrium frequency of the three-phase contact line motion ($K_{W}^{0}$) is obtained from Eyring's theory of absolute reaction rates [26]:

(4) $$\begin{array}{c} K_{W}^{0}=\left(\frac{k_{B}}{\hbar }\right)\exp\left(\frac{-\Delta G_{W}^{*}}{Nk_{B}T}\right)\;\\ \;\left(\mathrm{At}\;\mathrm{equilibrium}\;K_{W}^{0}=K^{+}=K^{-}\;\right) \end{array}$$

where $k_{B},\hbar ,N$,and *T* are the Boltzmann constant, Planck constant, Avogadro's number, and absolute temperature, respectively. In this equation, $\Delta G_{W}^{*}$ is the equilibrium free energy of capillary flow. An imbalance of the three interfacial tensions near the three-phase contact line, solid–liquid (*σ*_sl_), solid-vapor (*σ*_sa_), and liquid–vapor (*σ*), results in the out-of-equilibrium interfacial energy (*σ*(cos *θ*^0^ − cos *θ*)) which changes the total free energy of capillary flow. The frequency of the three-phase contact line motion in forward direction (+) and backward direction (−) is [26]:

(5) $$K^{\pm }=\left(\frac{k_{B}}{\hbar }\right)\;\exp\;\left(\frac{-\Delta G_{W}^{*}}{Nk_{B}T}\pm \frac{\sigma \left(\cos\theta^{0}-\cos\theta \right)}{2nk_{B}T}\right)$$

where *n* is the number of adsorption sites per unit area on solid surface. The net frequency of contact line motion is then as follows [26]:

(6) $$\begin{array}{l} K_{W}=K^{+}-K^{-}\\ \;=\left(\frac{2k_{B}}{\hbar }\right)\;\exp\;\left(\frac{-\Delta G_{W}^{*}}{Nk_{B}T}\right)\;\sinh\;\left(\frac{\sigma \left(\cos\theta^{0}-\cos\theta \right)}{2nk_{B}T}\right) \end{array}$$

For small arguments of sinh, Equations 3 and 6 result in linear MKT [31]:

(7) $$\sigma \left(\cos\theta^{0}-\cos\theta \right)=\mathrm{\zeta U}$$

where $\zeta =nk_{B}T/K_{W}^{0}\lambda$ is in units of Pa s and is termed as the coefficient of friction at the three-phase contact line. It is noted that this equation is identical to equation twenty-two of [33] for *U* = 0 and *σ* cos(*θ*^0^) = *σ*_sa_ − *σ*_sl_ (Young's equation). Left hand side (LHS) of Equation 7 is the out-of-equilibrium interfacial energy which is the driving force of capillary flow. Right hand side (RHS) of Equation 7 only includes dissipation of the free energy due to the contact line friction. De Ruijter et al. [30] showed that the corresponding dissipation function (*TΣ*_*l*_) is:

(8) $$T\Sigma_{l}=\frac{\zeta U^{2}}{2}$$

In the next section, the wedge film viscous dissipation is calculated and added to Equation 8 to form the total dissipation function from which the total drag force is calculated. The total drag force is then equated to the LHS of Equation 7 to form the complete equation of the three-phase contact line motion.

#### Hydrodynamic theory

To calculate the wedge film viscous dissipation (*TΣ*_*W*_), Navier–Stokes equation of motion is solved in the wedge film region. From Figure 4 for the film thickness (*H*) much smaller than the radial distance *ρ* (*H* ≪ *ρ*) and for capillary number Ca ≪ 1, lubrication theory is used:

(9) $$\frac{\partial p}{\partial x}=\frac{\partial }{\partial z}\left(\eta_{n}\frac{\partial u}{\partial z}\right)$$

where *p* is the pressure and *u* is the velocity distribution at distance *x* inside the wedge film. For no stress boundary condition at the free fluid-air interface and no slip boundary condition at the solid surface, solution to Equation 9 gives:

(10) $$u=\frac{1}{{\left(\eta_{b}F\left(\phi \right)K\right)}^{\frac{1}{n}}}{\left(\frac{\partial p}{\partial x}\right)}^{\frac{1}{n}}\frac{n}{n+1}\left(H^{\frac{1}{n}+1}-{\left(H-z\right)}^{\frac{1}{n}+1}\right)$$

where *η*_*n*_ is replaced by its expression in Equation 1. The average cross-sectional fluid velocity in the wedge film ($\bar{u}=\int_{0}^{H}\mathrm{udz}/H$) is equal to the three-phase contact line velocity ($\bar{u}=U$). This results in:

(11) $$u=\frac{2n+1}{n+1}U\left(1-{\left(1-\frac{z}{H}\right)}^{\frac{1}{n}+1}\right)$$

The viscous dissipation in the wedge film can be obtained as follows [5]:

(12) $$\begin{array}{l} T\sum_{W}=\int_{0}^{r-x_{m}}\left[\int_{0}^{H}\tau \left(\frac{\partial u}{\partial z}\right)\mathrm{dz}\right]\;\mathrm{d\rho}\\ \;=\eta_{b}F\left(\phi \right)K{\left(\frac{2n+1}{n}\right)}^{n}U^{n+1}\left\{\int_{0}^{r-x_{m}}\left[{\left(\frac{1}{H}\right)}^{n}\right]\mathrm{d\rho}\right\} \end{array}$$

where *τ* is the shear stress (= *η*_*n*_ ∂ *u*/∂ *z*), and *x*_*m*_ is the cutoff length similar to slip length in HDT [27,28]. Without consideration of *x*_*m*_, dissipation of energy at the wedge film grows infinitely close to the three-phase contact line. For a thin wedge film, solution to Equation 12 gives the wedge film viscous dissipation function:

(13) $$T\sum_{W}=\eta_{b}F\left(\phi \right)K{\left(\frac{2n+1}{n}\right)}^{n}U^{n+1}\frac{1}{\theta^{n}}\left[\frac{r^{1-n}-x_{m}{}^{1-n}}{1-n}\right]$$

#### Dynamic contact angle

Combining Equations 8 and 13 gives the total dissipation function [30]:

(14) $$T\Sigma_{l}+T\Sigma_{W}=\frac{\zeta U^{2}}{2}+\eta_{b}F\left(\phi \right)K{\left(\frac{2n+1}{n}\right)}^{n}U^{n+1}\frac{1}{\theta^{n}}\left[\frac{r^{1-n}-x_{m}{}^{1-n}}{1-n}\right]$$

Taking derivative of the total dissipation function with respect to contact line velocity (∂ [*TΣ*_*l*_ + *TΣ*_*W*_]/∂ *U*) results in the total drag force [5]:

(15) $$f_{\mathrm{drag}}=\mathrm{\zeta U}+\eta_{b}F\left(\phi \right)K{\left(\frac{2n+1}{n}\right)}^{n}\left(\frac{1+n}{1-n}\right)\frac{1}{\theta^{n}}\left[r^{1-n}-x_{m}{}^{1-n}\right]U^{n}$$

Finally, equating Equation 15 with LHS of Equation 7 gives:

(16) $$\begin{array}{l} \sigma \left(\cos\theta^{0}-\cos\theta \right)=\mathrm{\zeta U}\\ \;+\eta_{b}F\left(\phi \right)K{\left(\frac{2n+1}{n}\right)}^{n}\left(\frac{1+n}{1-n}\right)\frac{1}{\theta^{n}}\\ \;\times \left[r^{1-n}-x_{m}{}^{1-n}\right]U^{n} \end{array}$$

It is noted that for *n* = 1 (Newtonian fluid), the integral of Equation 12 results in logarithm ln(*r*/*x*_*m*_). In this case the final form of Equation 16 is similar to De Ruijter's model [30] (*σ*(cos *θ*^0^ − cos *θ*) = *ζU* + 6*ηΦ*(*θ*)*U* ln(*r*/*a*)) where *Φ* = sin ^3^*θ*/2 − 3 cos *θ* + cos ^3^*θ* and *a* is the cutoff length in De Ruijter's model).

In Equation 16, the base radius (*r*) is in millimeter length scale while the cutoff length (*x*_m_) is in nanometer length scale. Thus, *r* ≫ *x*_*m*_, and consequently *r*^*1*−*n*^ ≫ *x*_*m*_^*1−n*^ for *n* ranging from 0.04 to 0.92 (see Table 1). Also, for a sessile droplet of spherical geometry (see Figure 2), the base radius is geometrically related to the dynamic contact angle:

(17) $$r={\left(\frac{3V}{\pi }\frac{\sin^{3}\theta }{2-3\cos\theta +\cos^{3}\theta }\right)}^{\frac{1}{3}}$$

where *V* is the volume of the droplet. Neglecting *x*_m_^1 − *n*^ and substituting *r* with Equation 17 gives:

(18) $$\begin{array}{l} \sigma \left(\cos\theta^{0}-\cos\theta \right)=\\ \mathrm{\zeta U}+\eta_{b}F\left(\phi \right)K{\left(\frac{2n+1}{n}\right)}^{n}\\ \;\left(\frac{1+n}{1-n}\right)\frac{{\left(\frac{3V}{\pi }\frac{\sin^{3}\theta }{2-3\cos\theta +\cos^{3}\theta }\right)}^{\frac{1-n}{3}}U^{n}}{\theta^{n}} \end{array}$$

Equation 18 shows the dynamic contact angle (*θ*) as a function of contact line velocity (*U*), solid–liquid molecular interactions (*ζ*), and non-Newtonian viscosity (*n*, *K*). Finally, substituting *U* with *dr*/*dt* = (*dr*/*dθ*) × (*dθ*/*dt*) the following equation can be obtained for the time evolution of the dynamic contact angle:

(19) $$\begin{array}{l} \sigma \left(\cos\alpha -\cos\alpha^{0}\right)=\zeta \left[{\left(\frac{3V}{\pi }\frac{{\left(1+\cos\alpha \right)}^{6}}{{\left(2+3\cos\alpha -\cos^{3}\alpha \right)}^{4}}\right)}^{\frac{1}{3}}\right]\frac{\mathrm{d\alpha}}{\mathrm{dt}}\\ \;+\eta_{b}F\left(\phi \right)K{\left(\frac{2n+1}{n}\right)}^{n}\left(\frac{1+n}{1-n}\right)\left[{\left({\left(\frac{3V}{\pi }\frac{\sin^{3}\alpha }{2+3\cos\alpha -\cos^{3}\alpha }\right)}^{\frac{1}{3}}\right)}^{1-n}\right]\\ \;\times {\left[{\left(\frac{3V}{\pi }\frac{{\left(1+\cos\alpha \right)}^{6}}{{\left(2+3\cos\alpha -\cos^{3}\alpha \right)}^{4}}\right)}^{\frac{1}{3}}\right]}^{n}{\left(\frac{\mathrm{d\alpha}/\mathrm{dt}}{\pi -\alpha }\right)}^{n} \end{array}$$

in which the dynamic contact angle *θ* = *π* − *α*. To compare with experimental data *θ* is used. Equation 19 is an implicit ordinary differential equation, which cannot be solved analytically, and thus numerical solutions to this equation will be sought.

## Results and discussion

The effective diameter of nanoparticles was equal to 260 nm at the lowest solution concentration of 0.05 vol.%. At higher particle concentrations, the increased interparticle interactions result in larger clusters. This increases the possibility of clusters to deposit on the surface of solid and form a new hydrophilic surface. Due to their larger size, these clusters are less possible to deposit on the three-phase contact line, and thus a heterogeneous surface will form: within the wedge film and away from the three-phase contact line, deposition of TiO_2_ clusters results in a hydrophilic surface with higher surface energy (approximately 2.2 J/m^2^[34]) than the three-phase contact line where the bare borosilicate glass is present (approximately 0.11 J/m^2^[35]). The higher surface energy inside the droplet shrinks the wetted area by increasing the equilibrium contact angle (denser solutions are more hydrophilic inside than outside). As a result, solid–liquid interfacial tension increases which on the other hand enhances the equilibrium contact angle [21]. Surface tension of these solutions decreases with particle concentration that is in accordance with Gibb's adsorption isotherm. The shear thinning viscosity of the solutions is due to strong interparticle interaction of the nanoparticle clusters [19,23,36]. Other nanofluids such as ethylene glycol-based ZnO nanofluid [23] and CuO nanofluid [37] also exhibited shear thinning viscosity at low shear rates.

**Figure 5:**
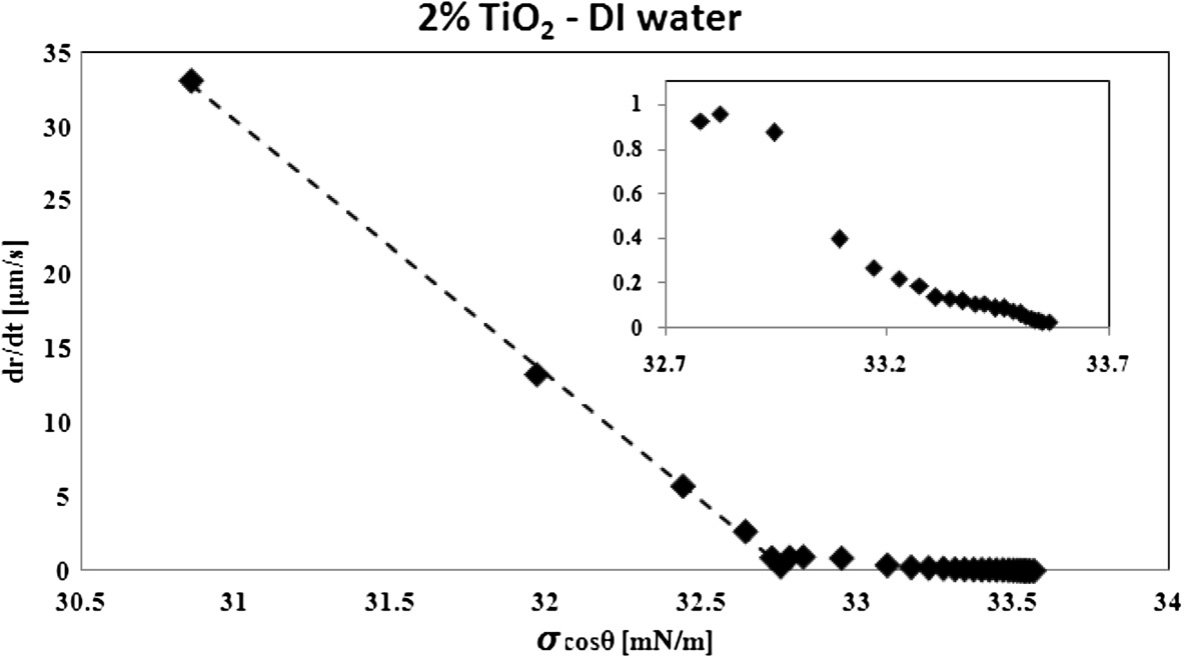
**Experimental three-phase contact line velocity (*U *= *dr*/*dt*) plotted versus *σ *cos *θ*.**

**Figure 6:**
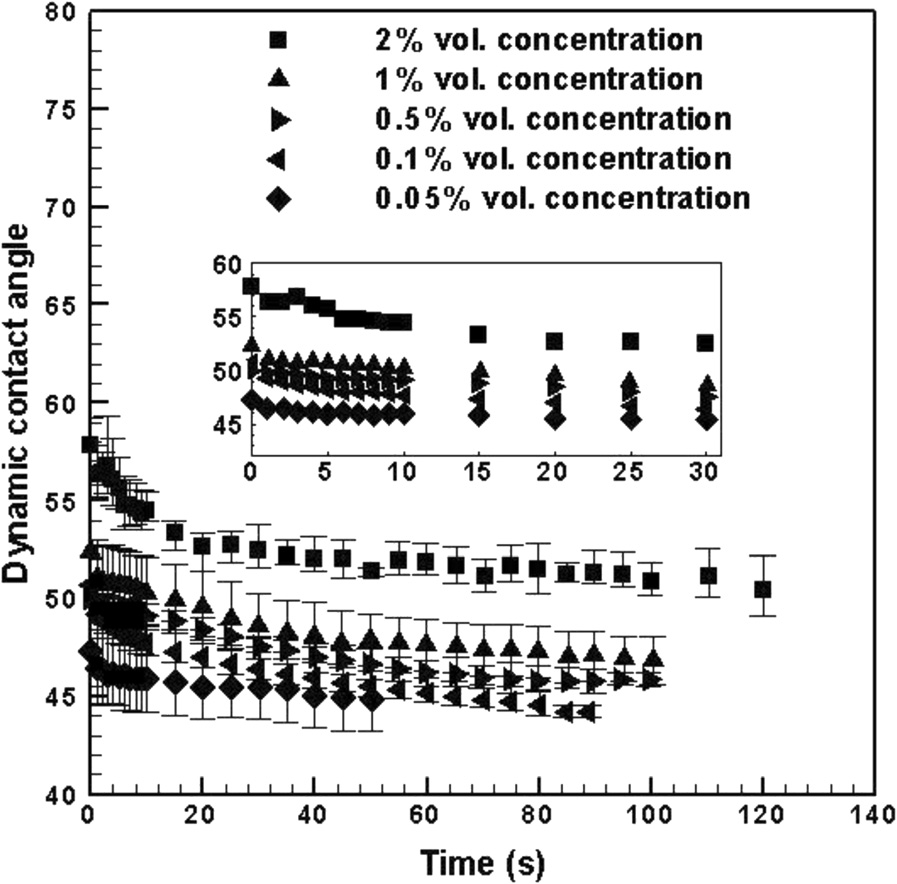
**Dynamic contact angle of TiO**_**2**_**-DI water solutions.**

Equation 19 suggests that the contact line friction dissipation (first term on the RHS of Equation 19) and the wedge film viscous dissipation (second term on the RHS of Equation 19) can occur at different time scales [38]. The time dependence of these dissipations has been shown by our experimental data: Figure 5 shows the experimental three-phase contact line velocity (*U* = *dr*/*dt*) plotted versus *σ* cos *θ*, where the base radius *r* is calculated from the experimental dynamic contact angle *θ* using Equation 17. Figure 5 shows a linear trend that is in accordance with the contact line friction dissipation and a nonlinear trend (see inset of Figure 5) that is in accordance with the wedge film viscous dissipation. This suggests that at the start of capillary flow, the contact line friction is the dominant dissipative mechanism. As capillary flow slows down, the wedge film viscous dissipation becomes more dominant. This corresponds to the solution's higher viscosity at lower shear rates (see Figure 3). Transition to wedge film viscous dominant regime occurs earlier in dilute solutions; for example, Figure 6 shows that for 0.05% concentration the viscous forces start to dominate at time scales around 4 to 8 s while for 2% concentration at time scales around 25 to 32 s.

Figure 6 shows the dynamic contact angle of TiO_2_-DI water nanofluids at various nanoparticle volume concentrations ranging from 0.05% to 2%. Due to limitation in camera frame per second speed (30 fps), the onset of pendant droplet touching the surface of solid cannot be determined accurately. Hence, the time axis in Figure 6 was shifted to where all of the captured images were readable to the FTA200 software. From Figure 6, it is obvious that for higher nanoparticle concentrations, the contact angles are higher. Figure 6 also shows that the spreading of these nanofluids starts from a primary region where the contact angle changes rapidly followed by a region where the contact angle changes more gradually (note that in a very short period of time (less than 300 ms), the contact angle evolves from 180° at point of contact to angles that are readable to our software and are plotted in Figure 6 at the shifted zero time). In the primary region, the contact line friction dissipation predominates the wedge film viscous dissipation causing fast reduction in the contact angle; then the wedge film viscous dissipation controls the droplet spreading [31].

**Figure 7:**
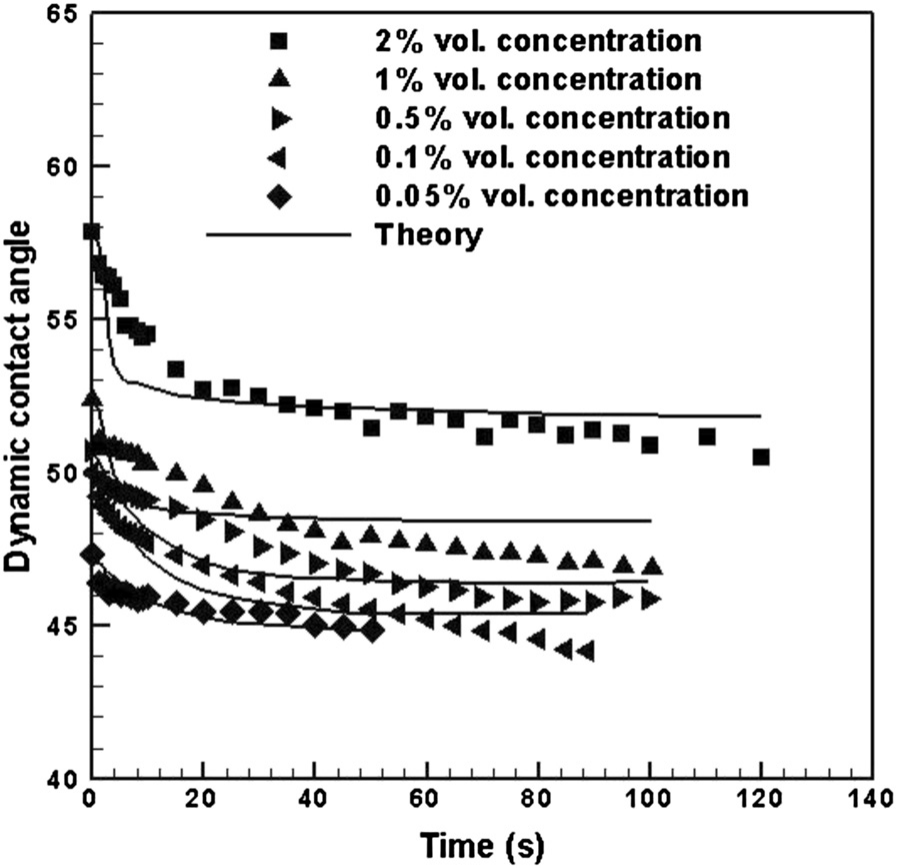
**Dynamic contact angle of TiO**_**2**_**-DI water nanofluid, comparison of experiment and theory.**

Using Equation 19, *ζ* is obtained for the best fit of theory to experimental data that gives the least squared error. Figure 7 shows a reasonable comparison between experimental data and theory. The error is calculated from the following equation and is reported in Table 2:

(20) $$\mathrm{error}=\sqrt{\frac{\sum_{i=1}^{N}{\left[\left(\theta_{i}^{\mathrm{fit}}-\theta_{i}^{\mathrm{experiment}}\right)/e\right]}^{2}}{N}}$$

where *N* is the number of experimental points, $\theta_{i}^{\mathrm{fit}}$ is the fitted dynamic contact angle, $\theta_{i}^{\mathrm{experiment}}$ is the experimental dynamic contact angle, and *e* = 1*°* is the experimental error associated with the standard deviation in the measurement of the contact angles. A close examination of table three in [31] and table four in [38] reveals that the agreement between experiment and theory in our case is reasonable considering the complexity of the solution.

**Table 2:** **Coefficient of contact line friction***ζ***, theoretical equilibrium contact angle**$\theta_{\mathrm{theory}}^{0}$, **and error of comparison between theory and experiment**

**Nanoparticle concentration**	***ζ*[Pa·s]**	$\theta_{\mathrm{theory}}^{0}$	**Error**
2%	32	52.1	1.1
1%	99	48.2	1
0.5%	464	46.4	0.65
0.1%	483	45.3	0.54
0.05%	486	44.8	0.34

Table 2 shows values of *ζ* for various nanoparticle volume concentrations. From solution concentration of 0.05% to 0.5% *ζ* only changes by 5%; however, it drops rapidly for denser solutions. It is possible that the relative higher hydrophobicity at the three-phase contact line for denser solutions lowers the affinity of surface molecules to water molecules, thereby lowering the friction. At dense concentrations, the presence of large amount of nanoparticles in the wedge film varies the flow field structure. Without nanoparticles, it has been stated that there are two flow patterns in the wedge film: rolling and lubricating patterns [5]. Nanoparticles in the wedge film can change these flow patterns and result in more complex flow structures. As a result of these interparticle interactions, dissipation is more pronounced in the wedge film. Equation 19 gives better results at lower nanoparticle concentrations since complex interparticle interactions are less frequent in dilute solutions (see Table 2). Other sources of disagreement between experiment and theory can be local variations in the concentration of the nanoparticles in the nanofluid [21], pinning of the contact line, and variations in solid–liquid interfacial tension (*σ*_sl_) [18,21]. It is not possible to model all these effects in theory, and only simple models which can accommodate some of these effects can be developed. Also shown in Table 2 are the theoretical equilibrium contact angles, $\theta_{\mathrm{theory}}^{0}$, which are in reasonable agreement with the experimental equilibrium contact angles, $\theta_{\exp}^{0}$ (see Table 1).

## Conclusions

Due to a wide range of industrial applications, studying capillary flow of liquids laden with metallic and metal oxide nanoparticles is important. Metal oxide TiO_2_ nanoparticles are especially interesting in enhanced heat removal applications. Agglomeration of nanoparticles results in clusters that have larger effective diameter than the actual particle size. These clusters can deposit on the surface of solid substrates and form a heterogeneous surface condition inside the droplet away from the three-phase contact line that increases the equilibrium contact angle. Dynamic contact angle of metal oxide TiO_2_ nanoparticles dispersed in DI water revealed two stages of spreading: rapid reduction in contact angle coincides with contact line friction dissipation governed by MKT while gradual reduction in contact angle coincide with wedge film viscous dissipation governed by HDT. Non-Newtonian viscosity of the solution is incorporated in HDT model to give reasonable comparison with experimental data. Nanoparticles in the wedge film change lubricating and rolling flow patterns and result in complex flow field structures. Including all physical aspects of such complex flow in theory is not feasible at the current stage. Simple theoretical equations can only give reasonable comparisons with experiment.

## Competing interests

The authors declare that they have no competing interests.
